# Bisphenol A in eggs causes development-specific liver molecular reprogramming in two generations of rainbow trout

**DOI:** 10.1038/s41598-017-13301-7

**Published:** 2017-10-26

**Authors:** Bastien Sadoul, Oana Birceanu, Neel Aluru, Jith K. Thomas, Mathilakath M. Vijayan

**Affiliations:** 1 Department of Biological Sciences, University of Calgary, Calgary, Alberta Canada; 2 Department of Biology, University of Waterloo, Waterloo, Ontario Canada; 3 Present Address: Biology Department, Woods Hole Oceanographic Institute, Woods Hole, MA USA

## Abstract

Bisphenol A (BPA) is widely used in the manufacture of plastics and epoxy resins and is prevalent in the aquatic environment. BPA disrupts endocrine pathways in fish, but the long-term developmental implications are unknown. We demonstrate that BPA deposition in the eggs of rainbow trout (*Oncorhynchus mykiss*), an ecologically and economically important species of fish, reprograms liver metabolism in the offspring and alters the developmental growth trajectory in two generations. Specifically, BPA reduces growth during early development, followed by a catch-up growth post-juveniles. More importantly, we observed a developmental shift in the liver transcriptome, including an increased propensity for protein breakdown during early life stages to lipid and cholesterol synthesis post- juveniles. The liver molecular responses corresponded with the transient growth phenotypes observed in the F1 generation, and this was also evident in the F2 generation. Altogether, maternal and/or ancestral embryonic exposure to BPA affects liver metabolism leading to development-distinct effects on growth, underscoring the need for novel risk assessment strategies for this chemical in the aquatic environment. This is particularly applicable to migratory species, such as salmon, where distinct temporal changes in growth and physiology during development are critical for their spawning success.

## Introduction

Water pollution is one of the major anthropogenic pressures on freshwater ecosystems, compromising its stability and sustainability and leading to species decline^1,2^. Industrial and domestic wastewater and agriculture effluents are the most relevant sources of contamination for aquatic ecosystems^2^. Bisphenol A (BPA), a widely used monomer to manufacture plastics and resins, is ubiquitous in the aquatic environment^3^, and concentrations as high as parts per million have been measured in global waterways^4^. Environmentally-realistic levels of BPA cause endocrine disrupting effects in aquatic species^5,6^, including invertebrates^7,8^, amphibians and fish^4,6^, suggesting possible ecosystem health impact by affecting various trophic levels.

In freshwater teleosts, chronic exposure to BPA during early development or throughout growth induces abnormal development, reproductive failure and disrupted metabolism^4^. Although most of these toxicological endpoints were observed at high BPA levels and in model organisms, including zebrafish (*Danio rerio*) and medaka (*Oryzias latipes*)^4,9,10^, more recent studies also support effects at environmentally relevant concentrations in non-model fish species, including carp (*Cyprinus carpio*) and brown trout (*Salmo trutta f*. *fario*)^5,11,12^. Moreover, BPA is maternally transferred in fish^13^ and this potentially could lead to long-term developmental effects even though the progeny was not exposed to water-borne BPA^14–16^. As BPA may bring about heritable epigenetic modifications^17^, early exposure to BPA may have long-term and multigenerational consequences in fish. For instance, a recent study showed that embryonic exposure of medaka (a model species with short generational time) to BPA led to transgenerational impact on reproductive performance and embryo survival^18^. However, little is known about multigenerational impacts of contaminants in non-model species, especially those with long generational times, such as salmonids.

Rainbow trout (*Oncorhynchus mykiss*) is an economically and ecologically important species widely distributed in North America. It is relevant for both its commercial and sports fisheries^19^ and, as a predator in its freshwater environment, it also plays a central role in the ecological network and ecosystem health^20^. We have previously shown that BPA accumulation in eggs affects growth and stress performance later in life, even though the chemical was no longer detected in the larvae at hatch^14–16^; however, we did not investigate whether the BPA effects are heritable in salmonids. Here we tested the hypothesis that BPA accumulation in eggs, mimicking maternal transfer of this contaminant, leads to long-term metabolic effects that are carried over to the next generation in rainbow trout, a species that usually reproduces at three years of age. We monitored the growth and changes in liver transcriptome (using RNA-Seq) over two generations in offspring raised from eggs containing BPA^15,16^. The development-specific transcriptome changes were assessed during the juvenile (~40 g) and post-juvenile (>40 g) growth phases of rainbow trout^21^. Our results indicate that maternal exposure to BPA and its deposition in eggs disrupts liver metabolic programming leading to distinct growth phenotypes that are life stage-specific and evident in at least two generations in rainbow trout.

## Results

### Bisphenol A (BPA) dynamics

**Figure 1:**
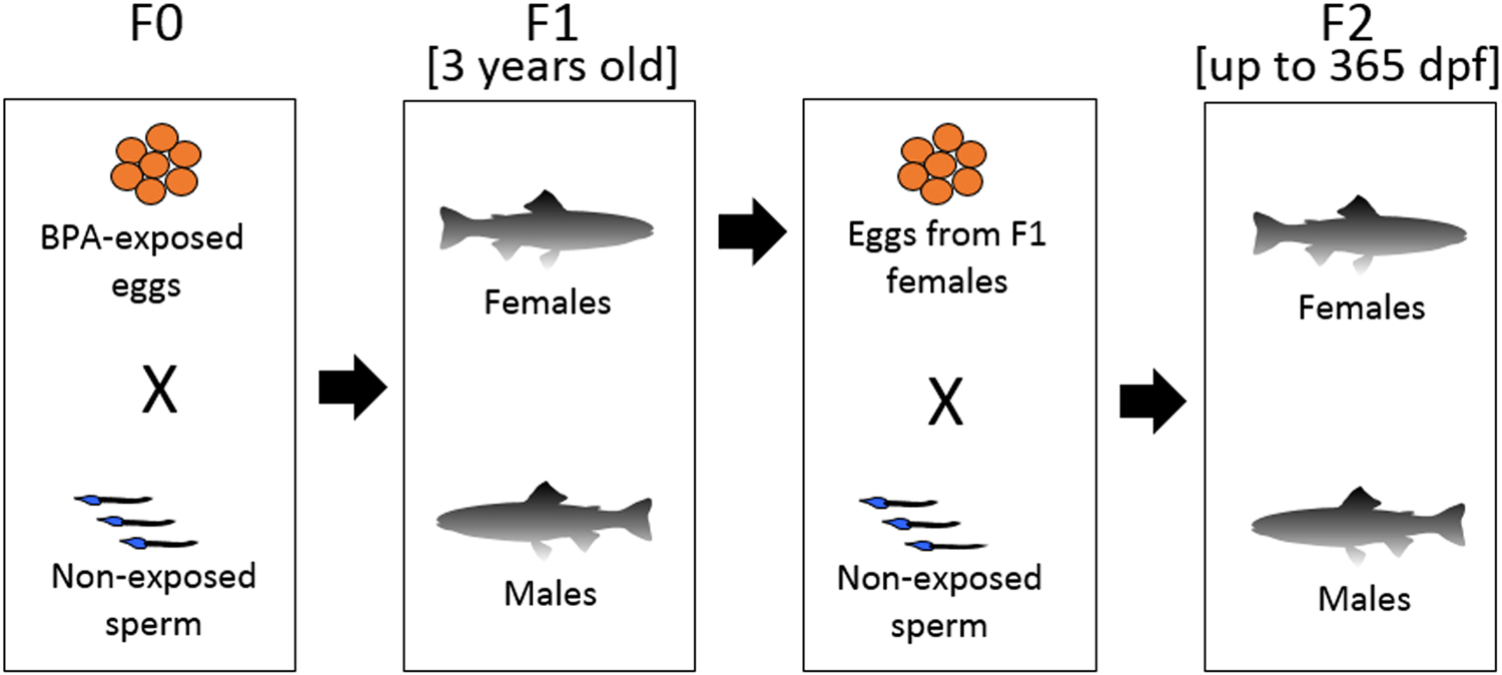
Two generation study design. F0 eggs were exposed to BPA in ovarian fluid for 3 h to mimic egg deposition of this contaminant as a result of maternal transfer. The eggs were fertilized with non-exposed sperm and reared at 8.5 °C until sexual maturity (3 years old). The F1 females raised from BPA-laden F0 eggs were stripped and the eggs were fertilized with the sperm of non-exposed males. The F2 larvae were reared in identical condition as F1 up to 365 days post fertilization.

Incubation of F0 eggs to 3 and 30 mg l^−1^ BPA for 3 h led to a net accumulation of 4.4 ± 0.9 and 41.3 ± 4.5 ng egg^−1^, respectively^16^. BPA depuration in embryos from fertilization to hatch has been previously reported^16^. As the exposure was specifically to enrich eggs to mimic a maternal BPA transfer scenario, the treatment is represented as 0 (Control), 4 (BPA4) and 40 (BPA40) ng egg^−1^ as described previously^16^. The accumulated BPA content in eggs steadily declined during embryogenesis and was below detection in all F1 groups at hatch (42 days post-fertilization; dpf)^16^. There were no effects of BPA accumulation in eggs on either hatch time or hatching success as reported previously^16^. Also, BPA was undetectable in eggs collected from 3 year old F1 females, raised from the BPA enriched eggs in F0 generation, from all treatments prior to fertilization with sperm from untreated males to produce the F2 generation (Fig. 1). Although there was no detectable BPA in eggs collected from F1 females, the primary germ cells giving rise to these eggs were exposed to the contaminant during embryogenesis.

### BPA accumulation in F0 eggs impacts growth

**Figure 2:**
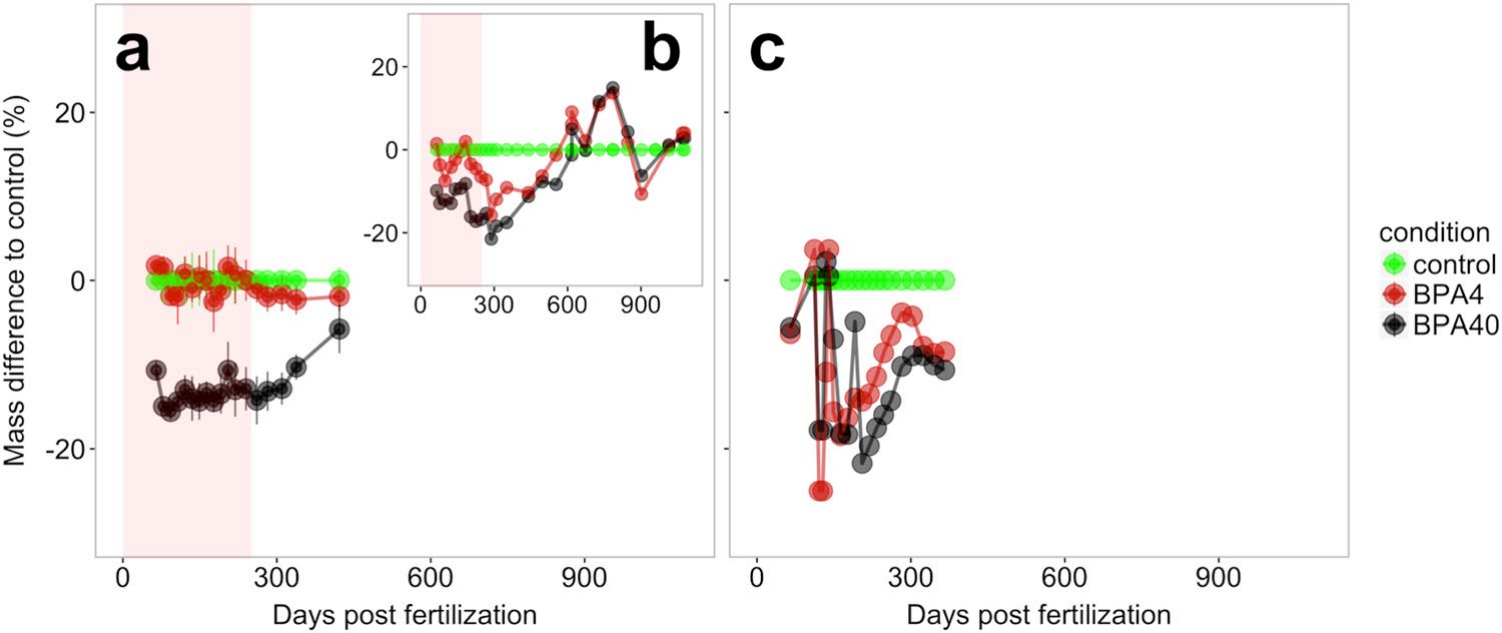
Bisphenol A in eggs affects the growth trajectory in rainbow trout. Difference in continuous growth from the control is plotted over time from two independent F1 generation studies [(**a**) and (**b**)] and from F2 generation (**c**) that were raised from eggs containing BPA at 4 (red graph) and 40 (black graph) ng per egg. BPA raised fish showed growth reduction until 260 dpf (red shade) followed by a catch-up growth over 365 dpf. Mean and standard error (n = 3) are shown for (**a**) because fish were reared in replicate tanks, whereas only one tank was used for the other studies. For each experiment, body mass difference to the control was calculated at each time and for each tank using the following equation: Mass difference to control = (Mass of tank-Mass of control)/Mass of control *100. With the mass of control being the mean body mass of control tanks at one time point.

The linear mixed model fitted to the first growth stage (0–260 dpf) in F1 generation showed a significant effect of treatment, but there was no effect of either time or interaction between time and treatment (Supplementary Table 1). Accumulation of 40 ng, but not 4 ng BPA egg^−1^ led to a significant (p = <0.001, z-value = −5.5) reduction in body mass (13%) relative to controls in the F1 generation over the first growth stage (0–260 dpf). This is illustrated by a constant weight difference between 40 BPA group and the control until 260 dpf observed in two independent studies (Fig. 2a and b). Model fit on the second growth stage (from 260 dpf) highlights significant effects of treatment, time and interaction (Supplementary Table 1). The significant interaction is the result of a positive slope over time for the high BPA group (p < 0.001, z-value = 8.553), which translates to a gradual, but significant reduction in the difference in body mass between the control and the BPA40 group (Fig. 2b). The growth trajectory trend with BPA in the F2 generation also appears similar to the F1 generation, but the lack of replicate tanks precludes any statistical analysis for the F2 generation (Fig. 2c). The individual growth metrics showed reduced weight and length in the BPA40 compared to the control at 140 dpf in the F1 (Supplementary Table 2), but no significant effects were observed between control and treatments either at 365 dpf in the F1 or at both developmental stages in the F2 (Supplementary Tables 2 and 3).

Proximate composition of the liver in the post-juveniles at 365 dpf indicated no significant differences in water, protein, carbohydrate and total energy contents in the F1 and F2 generations (Table 1). However, accumulation of 40 ng, but not 4 ng BPA per egg led to a significant (P = 0.017; z-value = 2.829) increase in total lipid content in the liver compared to the control group in both the F1 and F2 generations (Table 1).

**Table 1:** Liver proximate composition.

	Generation	Water Content (%)	Protein (%)	Carbohydrate (%)	Lipid (%)	Energy (kJ/g^−1^ of dried tissue)
**Liver**						
Control	**F1**	71.9 ± 0.6	12.7 ± 0.2	11.1 ± 0.8	4.3 ± 0.2	23.7 ± 0.2
4 ng BPA		74.1 ± 1.2	12.3 ± 1.1	8.9 ± 1.0	4.8 ± 0.2	24.5 ± 0.3
40 ng BPA		71.6 ± 0.6	12.8 ± 0.4	10.3 ± 0.9	5.3 ± 0.3	24.5 ± 0.4
Control	**F2**	74.9 ± 1.3	14.4 ± 0.6	6.1 ± 1.0	4.7 ± 0.2	25.2 ± 0.3
4 ng BPA		73.3 ± 1.4	13.2 ± 0.5	8.8 ± 1.9	4.7 ± 0.4	24.6 ± 0.6
40 ng BPA		74.0 ± 1.1	14.8 ± 0.5	6.0 ± 0.9	5.3 ± 0.4	25.5 ± 0.3
**Significant effect**		NS	NS	NS	*BPA40 > Control	NS

Trout liver proximate composition at 365 days post-fertilization in F1 and F2 generations (Mean ± SEM; n = 5–6). Significant effects are shown by an asterisk (2-way ANOVA followed by Dunnett’s test, p value < 0.05).

### BPA accumulation in F0 eggs impacts the liver transcriptome

**Figure 3:**
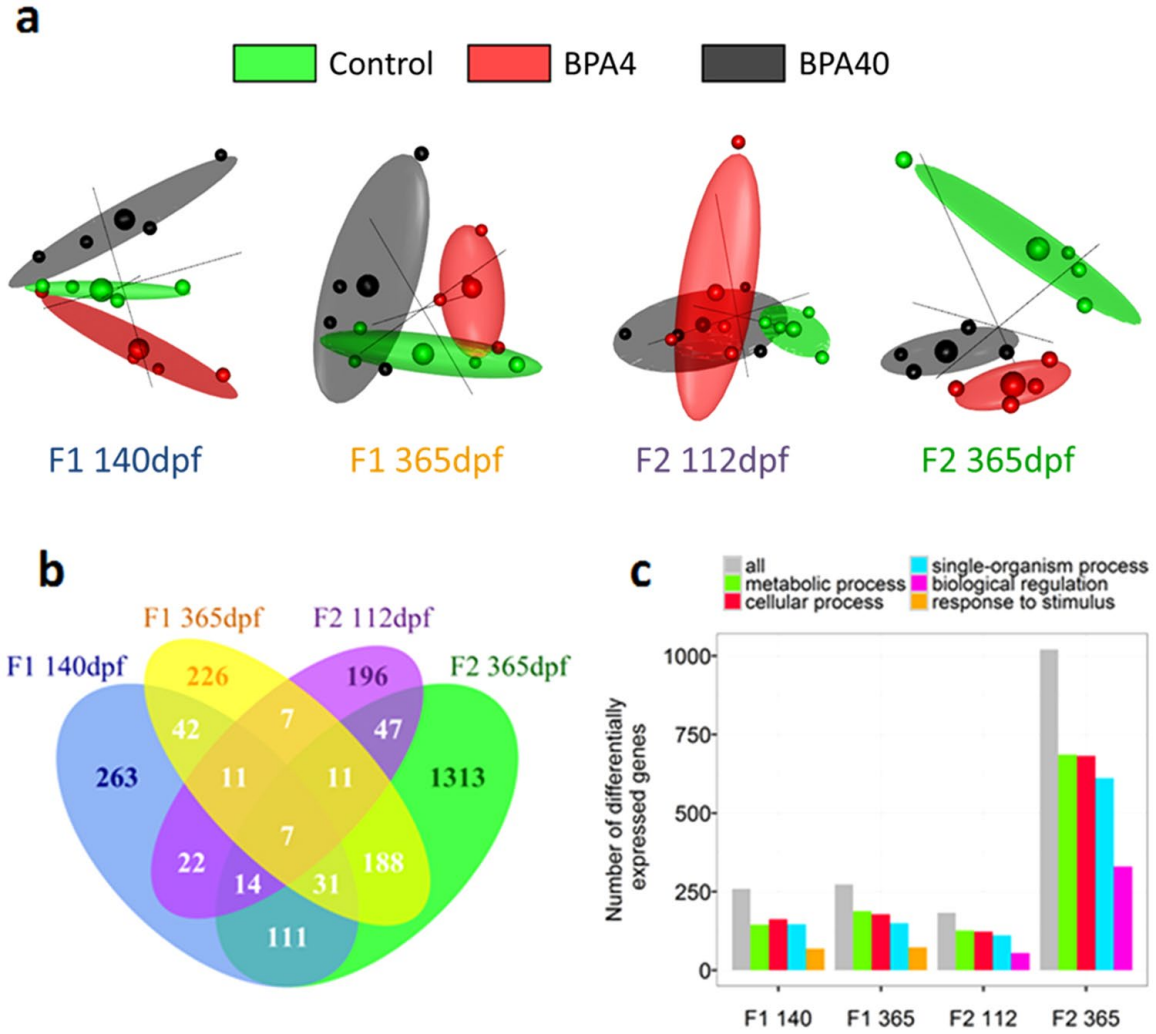
Liver transcriptome changes in trout raised from BPA enriched eggs. Liver transcriptome changes were assessed in response to BPA in eggs at either 140 or 112 dpf and 365 dpf in the F1 and F2 generations. (**a**) Three-dimensional Principal Component Analyses (PCA) on whole transcriptome for each generations and life stages. Expression of all the genes from the liver of individuals that were exposed to 0 (control, green), 4 (BPA4, red) or 40 (BPA40, black) ng BPA per egg at 140 dpf and 365 dpf in the F1 generation. Similarly liver transcript abundance from individuals obtained by breeding F1 females from the 3 groups with untreated males were used to create the PCA plots for each life stages in F2 generation (112 and 365 dpf). Small spheres represent one individual (n = 4), whereas the mean per treatment is represented by the bigger sphere and surrounded by the 3D ellipse of the standard error. (**b**) Venn diagram of differentially expressed genes between at least one BPA treatment and control for each time point. Genes differentially expressed between at least one BPA group and control were identified for each life stages and generations, and compared across stages and generations. (**c**) Enriched gene ontology terms among differentially expressed genes. Gene ontology enrichment analysis, using Blast2GO, was performed on all annotated genes differentially expressed in at least one BPA group and control.

Paired-end sequencing (Illumina) on RNA extracted from the liver of 4 fish per treatment over two generations (F1 and F2) and two life stages, resulting in a total of 48 samples, produced a total of 71,264,972 reads per sample with 96% mapped (Supplementary Table 4) to the 46,585 genes described in the recently published sequenced genome of rainbow trout^22^. A principal component analysis (PCA) was run for each time point using the whole transcriptome of every individual in each treatment. The 3D individual graph of the PCA illustrates the variability between the samples (Fig. 3a). The global expression variation among the samples showed a clear segregation pattern between the 3 treatments in F1 generation at 140 dpf and 365 dpf, and also in the F2 generation fish at 112 dpf and 365 dpf (Fig. 3a). These differences were confirmed by the identification of a total of 501, 523, 315 and 1722 differentially expressed genes (DEGs) between at least one BPA treatment and the control in F1 140 dpf, F1 365 dpf, F2 112 dpf and F2 365 dpf, respectively (Supplementary datasets). Many of these genes were shared between time points; 91 and 79 DEGs were shared between life stages within F1 and F2 generations, respectively (Fig. 3b). We also found 54 DEGs that were shared between F1 140 dpf and F2 112 dpf, and 237 that were shared between F1 365 dpf and F2 365 dpf (Fig. 3b). Based on Gene Ontology (GO) terms, 56, 69, 69 and 67% of the enriched genes were involved in metabolic processes (GO:0008152) in F1 140 dpf, F1 365 dpf, F2 112 dpf and F2 365 dpf, respectively (Fig. 3c, and Supplementary Tables 5–8). Among these genes involved in metabolic processes, genes related to protein metabolic process (GO:0019538) and lipid metabolic process (GO:0006629) were highly represented, including 289 and 92 DEGs, respectively, between the treatments in at least one time point (see below).

### BPA accumulation in F0 eggs impacts genes involved in protein metabolism

**Figure 4:**
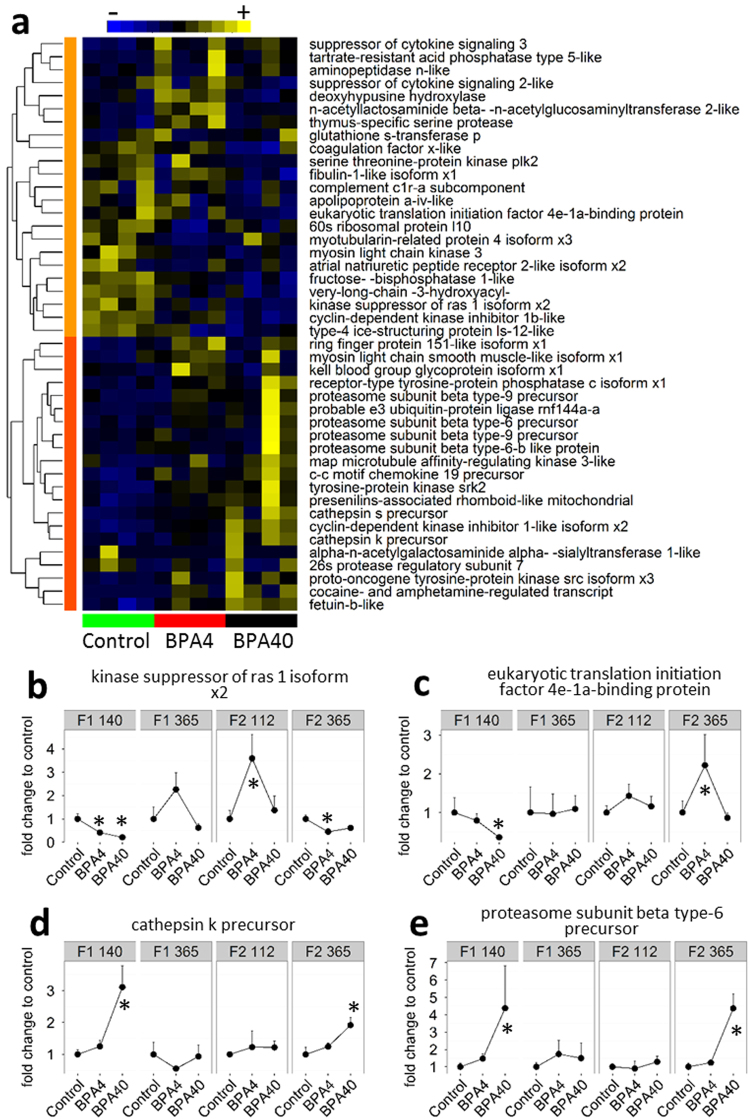
BPA in eggs affects protein metabolic processes. (**a**) Heat map of differentially expressed genes related to protein metabolic process in F1 140 days post-fertilization. All genes related to protein metabolic processes and differentially expressed in at least one of the BPA treatment are shown. For each gene, the expression level is indicated using a color density scale. Yellow and blue are used for overexpression and underexpression, respectively, whereas black is used for median expression. Based on their expression profile, genes were separated into two clusters. Cluster 1 (light orange) encloses mainly genes downregulated and cluster 2 (dark orange) contains genes mainly upregulated in BPA groups. (**b**–**e**) Fold change expression relative to the control for each time point of selected genes from cluster 1 (**b**,**c**) and cluster 2 (**d**,**e**). Significant difference from the control as calculated by Cuffdiff (false discovery rate set at <0.05) are highlighted using an asterisk in figures (**b**–**e)**.

Hierarchical clustering of DEGs involved in protein metabolic process (GO:0019538) showed two major groups in F1 generation at 140 dpf (Fig. 4a). The first cluster contained 23 genes that were mainly downregulated in the 4 and 40 ng BPA groups. According to the biological processes GO, this cluster was enriched with genes involved in biological regulation (9 genes, GO:0065007) and biosynthetic processes (6 genes, GO:0009058). Two genes, kinase suppressor of ras 1 isoform x2 (KSR1) and eukaryotic translation initiation factor 4e-1a-binding protein (4EBP), involved in the intracellular signaling pathways regulating the protein synthesis/proteolytic balance were part of this cluster and plotted for all time points in Fig. 4b and c. KSR1 (Fig. 4b) and 4EBP (Fig. 4c), were both significantly downregulated in the BPA40 group compared to the control in F1 at 140 dpf, but not at 365 dpf. KSR1 was also downregulated in BPA4 group in F1 140 dpf compared to the control. 4EBP was not significantly different from the control in all other time points except between BPA4 group and control in F2 generation where a 2-fold increase in the expression was observed (Fig. 4c). KSR1 was significantly upregulated and downregulated in the BPA4 group in F2 at 112 dpf and 365 dpf, respectively. In addition, genes playing a key role in protein synthesis, including 60S ribosomal protein l10 and myosin light chain kinase 3 were also found to be downregulated in the first cluster. The second cluster contained 21 genes upregulated in either BPA4 or BPA40 groups (Fig. 4a). This cluster was strongly enriched with genes involved in proteolytic processes (8 genes, GO:0006508). Most of these genes are part of the two most important proteolytic systems: the autophagy-lysosome system (ALS) for cathepsin s and k precursors and the ubiquitin-proteasome system (UPS) for proteasome subunits. The expression profiles of genes involved in proteolysis, including cathepsin k precursor (part of the ALS system) and proteasome subunit beta type-6 precursor (part of the UPS) were plotted for all time points (Fig. 4d and e). Both were significantly upregulated in the BPA40 group at 140 dpf but not at 365 dpf in the F1 generation. In the F2 generation both these genes were not significantly different in the BPA groups at 112 dpf, but they were significantly upregulated at 365 dpf in the BPA40 group (Fig. 4d and e).

In F1 365 dpf, hierachical clustering separated the genes related to protein metabolic process into 2 clusters (Supplementary Fig. 1). The first cluster contains 19 genes mostly upregulated for BPA groups and enriched for biosynthetic process (6 genes, GO:0009058). The second cluster has 33 genes downregulated in BPA groups and enriched for protein ubiquitination (10 genes, GO:0016567) and proteolysis (6 genes, GO:0006508). In F1, 365 dpf, protein synthesis was upregulated in BPA treated eggs and proteolysis downregulated (Supplementary Fig. 1).

In F2, 33 out of the 39 DEGs were part of a cluster of genes showing an upregulation in BPA groups at 112 dpf (Supplementary Fig. 2). Similarly to F1 generation 140 dpf, many of these genes (13) were part of the proteolytic process (GO:0006508). In contrast, 6 genes were downregulated in BPA groups, 2 being part of the biosynthetic process (GO:0009058). In F2, 365 dpf, 54 genes enriched for proteolysis (GO:0006508) were downregulated in BPA groups (Supplementary Fig. 3). In addition, 14 genes involved in biosynthetic process (GO:0006508) were downregulated in BPA treated groups (Supplementary Fig. 3).

### BPA accumulation in F0 eggs impacts the expression of genes related to lipid metabolism

**Figure 5:**
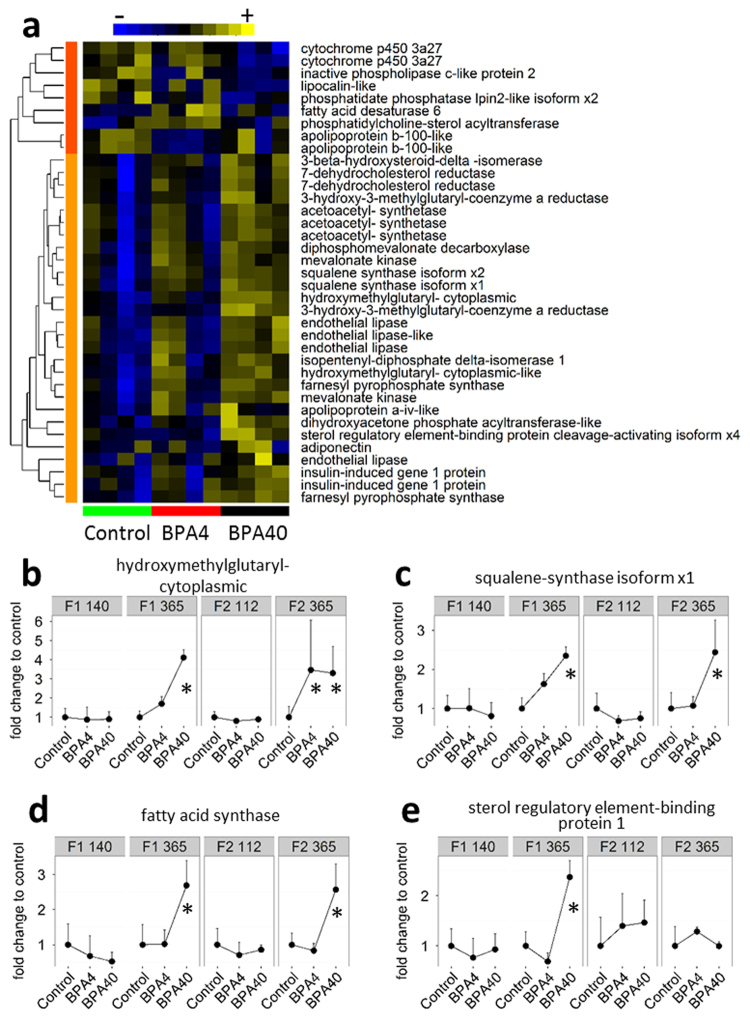
BPA in eggs affect lipid metabolic processes. (**a**) Heat map of differentially expressed genes related to lipid metabolic process in F1 365 days post fertilization. All genes related to lipid metabolic process and differentially expressed in at least one of the BPA treatment are shown. For each gene, the expression level is indicated using a color density scale. Yellow and blue are used for overexpression and under expression, respectively, whereas black is used for median expression. Based on their expression profile, genes were separated in two clusters. Cluster 1 (dark orange) mainly encloses genes downregulated in BPA groups, whereas cluster 2 (light orange) contains the genes mainly upregulated in BPA groups. (**b**–**c**) Fold change expression to the control for each time point of selected genes from cluster 2. (**d**–**e**) Fold change expression to the control for each time point of fatty acid synthase and sterol regulatory element-binding protein 1. Significant difference from the control as calculated by Cuffdiff (false discovery rate set at <0.05) are highlighted using an asterisk in figures (**b**–**e)**.

In F1 140 dpf, only 10 genes involved in lipid metabolic process (GO:0006629) were differentially expressed, 6 downregulated and 4 upregulated in BPA groups compared to the control (Supplementary Fig. 4). In F1 365 dpf, hierarchical clustering of DEGs related to lipid metabolism showed two major clusters (Fig. 5a). The first cluster contains 9 genes downregulated in the BPA groups, whereas the second cluster is grouping 28 genes upregulated in the BPA groups. In the second cluster, 17 genes are enriched for the biosynthesis process (GO:0009058). Specifically, 8 genes related to the cholesterol biosynthetic pathway (Supplementary Fig. 5a) are upregulated in BPA treatments (Fig. 5a). Concomitantly, cytochrome p450 3a27 (CYP3A27), involved in the inhibition of cholesterol synthesis, is downregulated in BPA treatments. Two genes, hydroxymethylglutaryl cytoplasmic (HMGCS1) and squalene synthase isoform x1 (FDFT1), representative of the second cluster and playing key roles in cholesterol biosynthesis pathway (Supplementary Fig. 5) were plotted at every time point (Fig. 5b,c). HMGCS1 (Fig. 5b) was not differentially expressed in F1 140 dpf and F2 112 dpf, but was significantly upregulated in BPA40 groups in F1 365 dpf and F2 365 dpf, and in BPA4 group in F2 365 dpf (Fig. 5b and Supplementary Fig. 5). FDFT1 was significantly upregulated in BPA40 group for F1 365 dpf and F2 365 dpf (Fig. 5c and Supplementary Fig. 5). Additionally, fatty acid synthase (FAS) and sterol regulatory element-binding protein 1 (SREBP1), although not categorized as proteins involved in lipid metabolism by our automated annotation method, play key roles in lipid synthesis and were, therefore, presented in Fig. 5d and e. Both were found to be more than 2x more expressed in BPA40 group in F1. Accumulation of 40 ng of BPA in the egg had also a similar effect for FAS in the F2 generation (Fig. 5d).

At F2 112 dpf, similarly to F1 140 dpf, only 14 genes related to lipid metabolism were differentially expressed in BPA groups with 9 genes upregulated in BPA groups and 5 genes downregulated (Supplementary Fig. 6). At F2 365 dpf, 31 genes were upregulated mostly enriched for the biosynthetic process (14 genes, GO:0009058), whereas 24 genes were downregulated in at least one BPA group (Supplementary Fig. 7).

## Discussion

BPA in eggs prior to fertilization, mimicking a maternal transfer scenario, alters growth and liver transcriptome in two generations of rainbow trout. Although BPA accumulation in the eggs reached levels that were reported in feral fish tissues^23,24^, the embryos at hatch were free of BPA^16^. Despite this, fish raised from BPA-treated eggs, especially 40 ng BPA/egg, showed distinct development-related growth phenotypes, including reduced growth during early life stages and compensatory catch-up growth and lipid accumulation in the liver in older individuals. The developmental effects on the liver transcriptome of trout at two life-stages (112–140 and 365 dpf) in two generations, support a distinct shift in molecular programming, underscoring long-term disruptions in growth and metabolism associated with maternal and/or ancestral exposure to BPA in trout.

The reduction in growth seen in the F1 BPA40 progeny at 140 dpf corresponded with enhanced potential for protein degradation, including upregulation of genes encoding key proteins of the autophagy and the ubiquitin-proteasome systems in trout liver (Fig. 4). In addition, KSR1 and 4EBP, two proteins interacting with MAPK^25^ and mTOR^26^ signaling pathways involved in stimulating protein synthesis and inhibiting proteolysis, were downregulated. This suggests impairment in protein synthesis/proteolysis balance during early development as a result of BPA deposition in eggs. This is further supported by the reduced specific growth rate and the disruption of the growth hormone/insulin-like growth factor (GH/IGF) axis in these fish during early development^16^.

**Figure 6:**
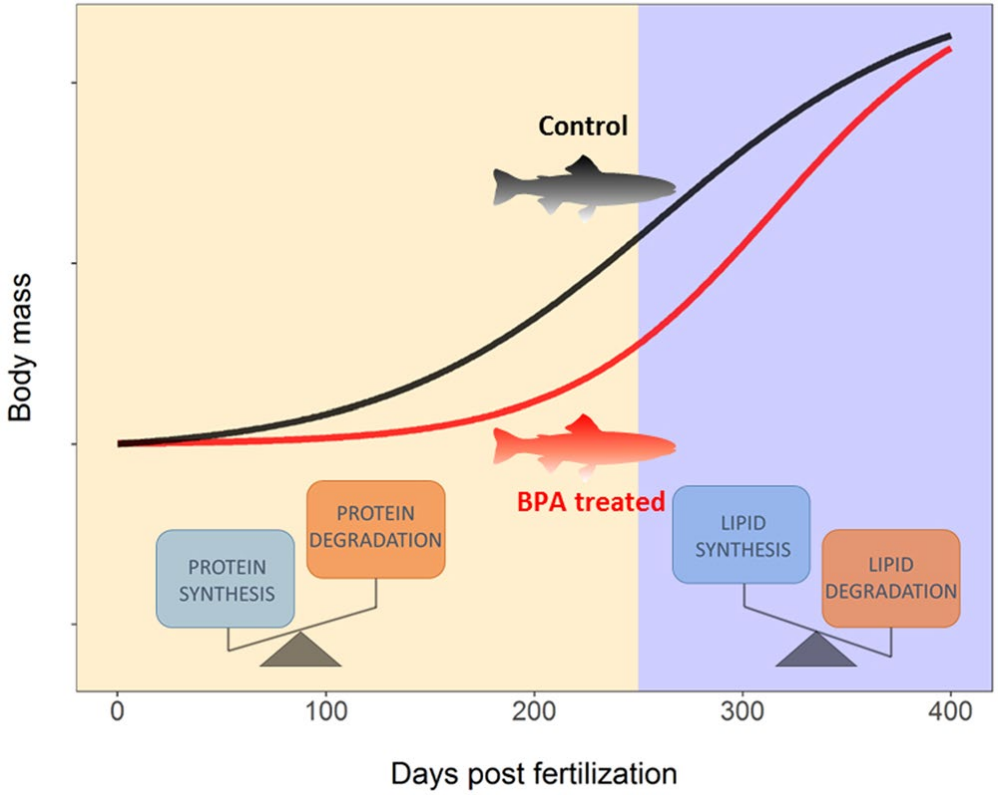
A conceptual model underpinning the developmental programming effects of BPA. Offsprings from BPA-laden eggs, but free of BPA after hatch, showed distinct shifts in long-term liver molecular programming and growth phenotypes. At early stages (up to 260 dpf), BPA treated fish show reduced body mass and this was associated with a shift towards increased protein degradation relative to protein synthesis. However, after 260 dpf, there was a catch-up growth in the BPA treated fish and this is associated with an increased lipid synthesis and protein synthesis relative to lipid breakdown.

Interestingly, the growth suppression seen in the BPA progeny during early life stages (140 dpf) were lost later in life (365 dpf) with the fish clearly showing a compensatory catch-up growth (Fig. 2). This developmental change in growth phenotype corresponded with a shift in liver metabolic machinery from proteolysis to protein synthesis (Fig. 4). Indeed, at 365 dpf, genes involved in the two proteolytic systems were downregulated, while genes involved in protein synthesis were upregulated (Supplementary Fig. 1). This temporal molecular shift in the protein synthesis/proteolysis balance supports the catch-up growth^27^ observed in BPA treated progeny after 260 dpf. The reason for the molecular shift favouring protein synthesis and reducing proteolysis in BPA groups after 260 dpf is unknown. However, the temporal catch-up growth shift coincides with the proposed changes in growth dynamics observed in post-juvenile rainbow trout^28^, which includes enhanced hypertrophy early on followed by hyperplasia post-juveniles. We propose that BPA in eggs is a developmental disruptor impacting stage-specific growth trajectories by affecting molecular mechanisms related to hyperplasia and hypertrophy in trout. The catch-up growth seen in BPA treated fish at the post-juvenile stage, along with the switch in the expression of genes related to the protein synthesis/proteolytic balance (Fig. 6a), supports disruption of developmental programming in trout.

Another key finding associated with the catch-up growth seen in BPA progeny was the enrichment of transcripts related to lipid metabolism. Specifically, key enzymes involved in fatty acid and cholesterol synthesis, including FAS, SREBP1 and squalene synthase were highly upregulated in BPA treated progeny at 365 dpf (Fig. 5) but not at 140 dpf. Additionally, we observed a decrease in the abundance of CYP3A27, a member of the CYP3A subfamily, playing an important role in the inhibition of cholesterol synthesis^29,30^. All these transcript changes support the notion that accumulation of BPA in the egg causes long-term aberrant liver lipid metabolism. More importantly, the observed transcript changes, including FAS and squalene synthase were persistent in the F2 generation pointing to liver lipid dysfunction in two generations. This notion finds support from the higher total lipid content in the liver of BPA progenies in both generations (Table 1). Higher lipid synthesis and cholesterol production, along with increased accumulation of fat in the liver, are common symptoms of obesity in animals^31^. Consequently, BPA deposition in eggs, mimicking a maternal transfer scenario, affects developmental programming in trout leading to a switch to a disrupted growth phenotype associated with liver lipid dysregulation, including upregulation of genes involved in cholesterol biosynthesis. More importantly, we show that the phenotype seen in the F1 generation was also evident in the F2 generation.

While multigenerational effects of BPA was evident in mice^32–34^, rats^35,36^, and medaka^18^, to our knowledge no study has examined the long-term impact of maternal transfer of contaminants on an ecologically and commercially important fish species. The high abundance of differentially expressed transcript in the F2 generation at 365 dpf was particularly telling (Fig. 3), and may entail an increased metabolic demand, impinging on other energy demanding but important pathways, including immune and reproductive processes. While the majority of studies have shown that BPA effects are ER- or ERR-mediated^37^, it is clear now that BPA may also have multiple modes of action independent of its xenoestrogenic effect^37^. To this end, recent studies demonstrated that BPA alters DNA methylation of several genes^38–40^, including genes involved in lipid metabolism in mice^41^. Also, micro RNAs has been implicated in the BPA-mediated epigenetic modifications^42^, and this effect can be inherited^43^. Consequently, the long-term changes seen in the liver transcriptome of progenies raised from BPA-laden eggs suggest epigenetic modifications as a plausible mechanism.

In conclusion, BPA in eggs, mimicking maternal transfer of contaminants, led to long-lasting liver metabolic dysfunction and affects the developmental growth trajectory in rainbow trout. The switch in the growth trajectory from reduced growth early on to an obesity phenotype is supported by the liver transcriptome changes in two generations. In both, F1 and F2, the protein synthesis/proteolysis balance in BPA groups is skewed towards proteolysis at early stages, but towards protein synthesis at 365 dpf, partially explaining the catch-up growth observed in BPA group between the two life stages (Fig. 6). Additionally, whereas no clear difference in the lipid synthesis/catabolism balance was observed between treatments at early stages, there was a clear stage-specific increase in lipid synthesis in the BPA treated progenies post-juveniles (Fig. 6), further supporting the catch-up phenomenon. Our results underscore long-term development-specific metabolic disorders associated with BPA deposition in trout eggs (Fig. 6). This may have ecological implications since growth and associated metabolism are crucial determinant of fitness, which ultimately drive foraging capacities, season-specific survival or predation vulnerability^44–46^. Also, given the genomic similarities between rainbow trout and other salmonids^47^, metabolic disruptions due to BPA may have serious conservation consequences^48^ in migratory species, including the Atlantic and Pacific salmons. In these species, the metabolic reserves, especially lipids and proteins^49,50^ need to be particularly well regulated in order to face the energetic cost of long-term migration linked to spawning. Any dysregulations in lipid and protein metabolism, as seen here in response to BPA exposure, may reduce the spawning success in these animals leading to long-term population declines^51,52^. Finally, our results raise new conceptual challenges in ecotoxicology modeling. Risk assessment of toxicant usually takes into account the tissue concentrations of a chemical, but our results reveal effects that are developmentally-distinct and occur even in the absence of any tissue contaminant level complicating our current understanding of risk assessment modelling. This is particularly applicable to migratory fish species, such as salmon, where distinct temporal changes in growth and physiology during development are critical for their spawning success and stock recruitment. We predict early exposure to BPA in these animals will disrupt developmental programming and may contribute to the population decline^51,52^.

## Materials and methods

### Experimental animals

Rainbow trout were bred and maintained at the Alma Aquaculture Research Station (AARS; Alma, Ontario, Canada), and all experiments were carried out in that facility as described previously^14,16^. The experimental protocols were approved by the Animal Care and Use Committees at the University of Guelph and the University of Waterloo and were in accordance with the Canadian council for animal care guidelines.

### Dosing of F0 eggs with BPA

The BPA dosing of eggs used in this study was published recently^15,16^. Briefly, eggs from four to six 3 + -year class rainbow trout females were pooled and equally distributed among three treatment groups, which were exposed to ovarian fluid supplemented with either vehicle (<0.01% ethanol), 3 mg l^−1^ BPA or 30 mg l^−1^ BPA. The oocytes were incubated for 3 h at 4–6 °C as described previously^14,16^, after which they were fertilized with 1–2 ml milt pooled from 4–6 males from the same brood stock as the females (Fig. 1). Water was added to activate the sperm and the embryos were washed with clean water and placed in Heath incubator trays receiving AARS well water (6–8 °C) at a rate of 10 l min^−1^. The subsequent embryos, larvae and adults were never exposed to BPA and were always maintained in clean water that had BPA levels below detection.

### F1 generation

F1 larvae were kept in the incubator trays for up to 49 dpf (one week post-hatch), after which they were moved to 200 l tanks receiving AARS well water at a rate of 10 l min^−1^ and a 12 h dark:12 h light photoperiod. We carried out two F1 studies, while experiment 1 did not have tank replication for treatments, experiment 2 was distributed into 3 tanks per treatment (n = 277–299 larvae per tank). From 65 dpf onwards fish were fed to satiation^14^. Body mass were estimated by measuring the total mass divided by the number of fish in the tank at 65, 79, 93, 107, 121, 135, 149, 163, 177, 191, 205, 219, 240, 261, 282, 310, 338 and 422 dpf. Mortality over this period in the BPA treatments was not significantly different from controls.

### F2 generation

Three year old F1 females from the above treatment groups were used to generate the F2 fish (Fig. 1) as previously described for the F0 generation^16^. Fertilized eggs were placed in the Heath chambers and maintained under identical conditions as the F1 generation at the AARS facility and fish were weighed and fed as described above. Only one tank per treatment was available for the longer-term maintenance of the F2 generation.

### Fish sampling

All samples for the F1 generation were obtained from experiment 2 (described above). A subset of fish was terminally sampled at 140 and 365 dpf in the F1 generation and at 112 dpf and 365 dpf in F2 generation. Food was withheld for 48 h prior to all sampling times and the animals were anesthetized with a lethal dose of methanetricaine sulfonate (MS222; 1.0 g l^−1^) buffered with sodium bicarbonate (2.0 g l^−1^). Pieces of liver were quickly collected and frozen on dry ice and stored frozen at −80 °C until transcriptome analysis.

### Analytical techniques

#### Proximate composition

Total protein content in the liver was determined by the bicinchoninic acid (BCA) method (Sigma-Aldrich, St. Louis, MO) and glucose and glycogen was measured colorimetrically according to established protocols^53^. Lipid content was determined by Folch method^54^, and the gross energy content was calculated by using values of 5.65, 9.45 and 4.2 kcal g^−1^ for protein, fats and carbohydrates, respectively^55^.

#### RNA extraction and next-generation sequencing

Four liver samples per treatment per time-point and per generation (4 x 3 treatment x 2 time-points x 2 generations = 48 samples) were used for transcriptome analysis. Total RNA was extracted using the RNeasy Mini Kit (Qiagen, Hilden, Germany), DNase treated according to the manufacturers’ recommendations and eluted in 30 uL of nuclease free water. The quantity and integrity of RNA was assessed using the Agilent RNA ScreenTape assay, with the Agilent 2200 TapeStation system (Agilent, Santa Clara, CA). RNA sequencing was carried out at the Alberta Children’s Hospital Research Institute at the University of Calgary.

RNA-Seq libraries were prepared using the Illumina TruSeq Stranded mRNA kit, following the standard protocol from Illumina. Next-generation sequencing (NGS) was performed on an Illumina NextSeq. 500 platform (Illumina Inc., San Diego, CA, USA) using paired-end reads v2 chemistry with 75 bp sequencing.

#### RNA-Seq data analysis

Raw reads were trimmed and filtered using Illumina base calling software bcl2fastq (2.14). Reads were then mapped to the recently published sequenced genome of rainbow trout^22^ obtained from http://www.genoscope.cns.fr/trout/. This was performed using Bowtie (2.2.6)^56^ with the “very-fast-local” option and Tophat (2.1.0)^57^. Fragments Per Kilobase of transcript per Million mapped reads (FPKM) calculation for each sample was performed by Cufflinks (2.2.1)^58^ with the “multi-read-correct” and “GTF-guide” options, as recommended when the genome is available for the species and paired-end sequencing was performed^59^. Finally, Cuffdiff, integrated in Cufflinks,was used to highlight significant differences between the treatments, with the false discovery rate set as <0.05. Following the methods described in the supporting information, the genes were then annotated and expression profiles were illustrated using PCA, heat-maps and, for individual genes, line plots of normalized expression levels to the control group.

#### Statistical analyses for mass over time, survival and proximate composition

All data analyses were performed using R 3.3.1 (http://cran.r-project.org/). Survival integrated over the entire experiment was compared between the treatments using an ANOVA. For each experiment, body mass difference to the control was calculated at each time and for each tank using the following equation:

Mass difference to control = (Mass of tank−Mass of control)/Mass of control *100.

With the mass of control being the mean body mass of control tanks at one time point.

Difference in body mass between treatments and control were fitted using a segmented regression model, which provided an estimated breakpoint for BPA40 at around 260 dpf. A linear independent analysis was performed on two growth stages: from 0 to 260 dpf and time-points after 260 dpf. At each stage, a linear mixed model was fitted with time, treatment and interaction used as fixed effects, while also taking into account the random effects due to tank and experiment. The model was then analysed with an ANOVA performed with lme from the nlme package based on Satterthwaithe approximated degrees of freedom^60^. Between treatment comparisons were performed using contrasts in the glht function from multcomp package in R^61^. Proximate composition was analysed for each measure using a 2-way ANOVA with treatment and generation as fixed effects. A Dunnett’s test was performed to compare treatments with control.

### Data availability

RNA-seq data has been deposited in a publicly accessible database (GEO repository, Accession #: GSE94281; http://www.ncbi.nlm.nih.gov/geo/query/acc.cgi?acc=GSE94281).

## Electronic supplementary material

Supplementary Information
