# Supplementary material for: Bisphenol A in eggs causes development-specific liver molecular reprogramming in two generations of rainbow trout
Source: Sci Rep. 2017 Oct 26;7:14131. doi: 10.1038/s41598-017-13301-7 (PMC5658357; doi:10.1038/s41598-017-13301-7)
Supplement: Supplementary file 1 — Supplementary Information [file 41598_2017_13301_MOESM1_ESM.pdf]

Supplemental information

Bisphenol A in eggs causes development-specific liver molecular reprogramming in two generations of rainbow trout

Bastien Sadoul[1], Oana Birceanu[2], Neel Aluru[2,3], Jith K. Thomas[1] and Mathilakath M. Vijayan[1,2,*]

[1]Department of Biological Sciences, University of Calgary, Calgary, Alberta, Canada, and [2]Department of Biology, University of Waterloo, Waterloo, Ontario, Canada
[3]*Present address:* Biology Department, Woods Hole Oceanographic Institute, Woods Hole, MA, USA

**Corresponding author and address:*

Dr. M.M. Vijayan
Department of Biological Sciences
University of Calgary,
Calgary, Alberta
T2N 1N4, Canada
E-mail: matt.vijayan@ucalgary.ca

Materials and methods

Gene annotation, and GO enrichment

Differentially expressed genes between control and at least one BPA treatment were annotated and enriched using Blast2GO with default settings (v.3.1.3) [1]. First, homolog search was performed using BLASTX (e-value of 10^{-3}) against the NCBI non-redundant protein (NR) database. Then, functional annotation was completed using InterPro search [2]. In total, this procedure assigned a Gene Ontology (GO) term to 74% of the differentially expressed genes. All annotations were compiled with the free software R 3.3.1 (http://cran.r-project.org/), in an annotation database package using the tool AnnotationForge from Bioconductor [3]. Functional profiles were described using package goProfiles from Bioconductor [4].

Visualization of transcriptomics data

Transcriptomic profiles were first compared between treatments using a Principal Component Analysis (PCA) ran on log-transformed FPKM values of the 46,585 genes for each time point and generation using the FactoMineR package in R [5]. The coordinates on the three first principal components were illustrated in a 3D graph with the plot3d function from rgl package [6]. The first 3 axes of the PCA represented, respectively, 22, 12 and 10% of the total variability for F1 140 dpf, 15, 14 and 12% for F1 365 dpf, 23, 13 and 11% for F2 112 dpf, and 22, 12, 11% for F2 365 dpf.

Expression profiles of genes enriched for the gene ontologies "protein metabolic process" (GO:0019538) and "lipid metabolic process" (GO:0006629) were illustrated in a heat-map for generation and time-point. The FPKM were log-transformed and standardized (reduced and centered) across all treatments and submitted to heatmap.2 function in gplots package [7] in R. Genes were separated in two main clusters using a hierarchical cluster analysis using the hclust function in R.

Pathway analysis

Network corresponding to the Cholesterol Biosynthesis pathway for *Homo Sapiens* (WP197) was imported into Cytoscape (v. 3.4.0). Genes associated by Blast2GO for lipid metabolism (GO:0006629) were queried for their closest human homolog gene ID in NCBI "Gene" database, using rentrez package in R [8]. Gene ID were then used as shared names in Cytoscape to highlight differentially expressed genes in the BPA40 group.

1. Götz, S. *et al.* High-throughput functional annotation and data mining with the Blast2GO suite. *Nucleic Acids Res.* **36,** 3420–3435 (2008).
2. Mitchell, A. *et al.* The InterPro protein families database: the classification resource after 15 years. *Nucleic Acids Res.* **43,** D213–D221 (2015).
3. Huber, W. *et al.* Orchestrating high-throughput genomic analysis with Bioconductor. *Nat. Methods* **12,** 115–121 (2015).
4. Sanchez, A., Ocana, J. & Salicru, M. *goProfiles: goProfiles: an R package for the statistical analysis of functional profiles*. (2016).
5. Lê, S., Josse, J. & Husson, F. FactoMineR: an R package for multivariate analysis. *J. Stat. Softw.* **25,** 1–18 (2008).
6. Adler, D. *et al.* rgl: 3D visualization using OpenGL. *R Package Version 095* **1247,** (2015).
7. Warnes, G. R. *et al.* gplots: Various R programming tools for plotting data. *R Package Version* **2,** (2009).
8. Winter, D. *rentrez: Entrez in R*. (2016).

Supplementary Table 1. ANOVA outputs from linear mixed models fitting the two growth stages. For each growth stage and variable, the numerator degrees of freedom (numDF), denominator degrees of freedom (denDF), F-value and p-value are provided.

Growth stage	Variables	numDF	denDF	F-value	p-value
1	Intercept	1	132	36.34814	<0.001
	Treatment	2	8	30.35022	<0.001
	Time	1	132	0.28178	0.596
	Treatment:Time	2	132	0.09337	0.911
2	Intercept	1	52	6.20831	0.016
	Treatment	2	8	16.52385	0.001
	Time	1	52	44.58282	<0.001
	Treatment:Time	2	52	18.51916	<0.001

Supplementary Table 2. Body characteristics of F1 generation rainbow trout at 140 and 365 dpf (n=221-225 at 140 dpf, n=48 at 365 dpf for each concentration). Data points sharing the same letter within one life stage are not statistically different (one-way ANOVA, Tukey's post-hoc test, $p<0.05$; an ANOVA on ranks was run when the data did not meet the requirements of normality).

		**BPA accumulation in oocytes prior to fertilization** (ng embryo^{-1})		
**Life stage**		**Control**	**4**	**40**
140 dpf	Length (cm)	6.24 ± 3.56^{a}	6.15 ± 3.02^{a}	5.90 ± 3.28^{b}
	Weight (g)	2.25 ± 0.04^{a}	2.16 ± 0.04^{a}	1.96 ± 0.03^{b}
	CF	0.91 ± 0.01^{a}	$0.92 \pm 0.01^{a,b}$	0.94 ± 0.01^{b}
	HSI	1.43 ± 0.15^{a}	1.52 ± 0.10^{a}	1.75 ± 0.19^{a}
365 dpf	Length (cm)	19.45 ± 0.3^{a}	19.02 ± 0.30^{a}	18.49 ± 0.34^{a}
	Weight (g)	81.14 ± 3.53^{a}	75.76 ± 3.12^{a}	70.98 ± 3.82^{a}
	CF	1.08 ± 0.03^{a}	1.07 ± 0.01^{a}	1.06 ± 0.01^{a}
	HSI	1.47 ± 0.13^{a}	1.32 ± 0.13^{a}	1.46 ± 0.14^{a}

Supplementary Table 3. Body characteristics of F2 generation rainbow trout at 112 and 365 dpf (n=10-14 at 112 dpf and n=24 at 365 dpf). Data points sharing the same letter within one life stage are not statistically different (one-way ANOVA, Tukey's post-hoc test, p<0.05; an ANOVA on ranks was run when the data did not meet the requirements of normality).

		Ancestral BPA accumulation in oocytes prior to fertilization (ng embryo^{-1})		
**Life stage**		**Control**	**4**	**40**
112 dpf	Length (cm)	3.77 ± 0.89^{a}	3.96 ± 0.84^{a}	3.93 ± 0.96^{a}
	Weight (g)	0.6 ± 0.03^{a}	0.73 ± 0.10^{a}	0.77 ± 0.04^{a}
	CF	1.13 ± 0.06^{a}	1.16 ± 0.14^{a}	1.29 ± 0.10^{a}
	HSI	1.74 ± 0.08^{a}	2.03 ± 0.22^{a}	1.72 ± 0.10^{a}
365 dpf	Length (cm)	19.73 ± 0.72^{a,b}	21.65 ± 0.41^{a}	19.47 ± 0.40^{b}
	Weight (g)	103.77 ± 10.74^{a,b}	126.92 ± 7.76^{a}	90.61 ± 5.65^{b}
	CF	1.23 ± 0.02^{a}	1.21 ± 0.03^{a}	1.19 ± 0.02^{a}
	HSI	0.95 ± 0.11^{a}	1.01 ± 0.08^{a}	1.04 ± 0.05^{a}

Supplementary Table 4. Number of reads and percentage mapped to the reference trout genome for each sample.

Generation	Days Post Fertilization	Treatment	Number of reads	Percentage m
F1	365	Control	66,954,638	
F1	365	Control	71,363,620	
F1	365	Control	65,353,052	
F1	365	Control	82,542,851	
F1	365	4 ng BPA	72,748,374	
F1	365	4 ng BPA	79,325,120	
F1	365	4 ng BPA	74,081,216	
F1	365	4 ng BPA	81,492,726	
F1	365	40 ng BPA	73,093,457	
F1	365	40 ng BPA	84,684,128	
F1	365	40 ng BPA	81,068,806	
F1	365	40 ng BPA	94,357,239	
F2	365	Control	92,452,116	
F2	365	Control	90,382,655	
F2	365	Control	86,108,198	
F2	365	Control	80,036,939	
F2	365	4 ng BPA	62,996,579	
F2	365	4 ng BPA	70,776,557	
F2	365	4 ng BPA	74,252,059	
F2	365	4 ng BPA	77,484,062	
F2	365	40 ng BPA	88,074,145	
F2	365	40 ng BPA	82,214,979	
F2	365	40 ng BPA	95,093,565	
F2	365	40 ng BPA	83,611,222	
F1	140	Control	64,147,301	
F1	140	Control	63,916,660	
F1	140	Control	67,970,003	
F1	140	Control	62,094,308	
F1	140	4 ng BPA	69,181,343	
F1	140	4 ng BPA	65,884,602	
F1	140	4 ng BPA	58,364,313	
F1	140	4 ng BPA	66,290,824	
F1	140	40 ng BPA	63,455,867	
F1	140	40 ng BPA	65,789,757	
F1	140	40 ng BPA	58,481,713	
F1	140	40 ng BPA	68,045,374	
F2	112	Control	63,457,844	
F2	112	Control	66,818,974	
F2	112	Control	52,170,499	
F2	112	Control	51,874,835	
F2	112	4 ng BPA	63,340,021	
F2	112	4 ng BPA	54,450,723	
F2	112	4 ng BPA	58,887,533	
F2	112	4 ng BPA	59,301,656	
F2	112	40 ng BPA	62,096,483	
F2	112	40 ng BPA	62,411,760	
F2	112	40 ng BPA	83,551,660	
F2	112	40 ng BPA	58,186,323	

Supplementary Table 5. Most represented Gene Ontology (GO) terms of biological processes of level 2 among differentially expressed genes (DEGs) for at least one BPA treatment compared to control in F1 generation 140 dpf.

GO TERM	NUMBER OF DEGs
cellular process	152
metabolic process	144
single-organism process	136
response to stimulus	69
biological regulation	68
localization	56
regulation of biological process	54
signaling	37
cellular component organization or biogenesis	34
developmental process	32
immune system process	29
multicellular organismal process	18
locomotion	12
negative regulation of biological process	10
biological adhesion	7
growth	6
positive regulation of biological process	6
multi-organism process	4

Supplementary Table 6. Most represented Gene Ontology (GO) terms of biological processes of level 2 among differentially expressed genes (DEGs) for at least one BPA treatment compared to control in F1 generation 365 dpf.

GO TERM	NUMBER OF DEGs
metabolic process	188
cellular process	176
single-organism process	145
response to stimulus	73
biological regulation	58
regulation of biological process	51
developmental process	47
cellular component organization or biogenesis	37
signaling	36
localization	35
immune system process	33
multicellular organismal process	21
growth	12
locomotion	8
multi-organism process	7
biological adhesion	6
negative regulation of biological process	2
positive regulation of biological process	2
reproduction	2
behavior	1

Supplementary Table 7. Most represented Gene Ontology (GO) terms of biological processes of level 2 among differentially expressed genes (DEGs) for at least one BPA treatment compared to control in F2 generation 112 dpf.

GO TERM	NUMBER OF DEGs
metabolic process	124
cellular process	113
single-organism process	101
biological regulation	55
regulation of biological process	50
response to stimulus	37
localization	35
signaling	17
cellular component organization or biogenesis	15
developmental process	12
negative regulation of biological process	12
positive regulation of biological process	12
immune system process	11
multicellular organismal process	9
growth	4
multi-organism process	3
biological adhesion	1
locomotion	1

Supplementary Table 8. Most represented Gene Ontology (GO) terms of biological processes of level 2 among differentially expressed genes (DEGs) for at least one BPA treatment compared to control in F2 generation 365 dpf.

GO TERM	NUMBER OF DEGs
metabolic process	659
cellular process	620
single-organism process	546
biological regulation	330
regulation of biological process	291
response to stimulus	268
localization	196
signaling	171
multicellular organismal process	107
developmental process	103
cellular component organization or biogenesis	97
immune system process	83
negative regulation of biological process	52
positive regulation of biological process	43
biological adhesion	34
locomotion	27
growth	19
multi-organism process	16
behavior	6
reproduction	4

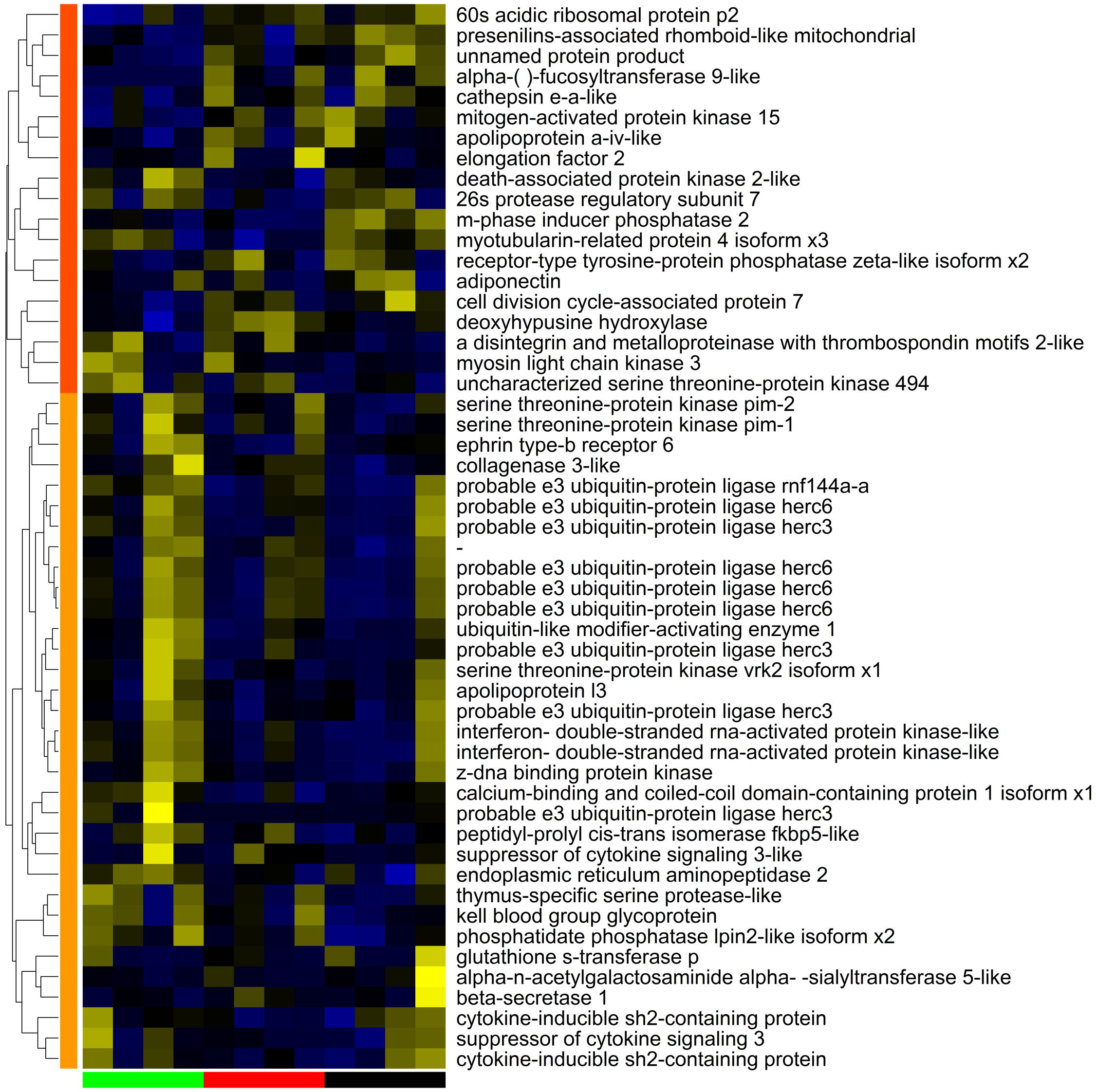

Supplementary Figure 1. Heatmap for genes related to protein metabolic process and differentially expressed between at least one BPA treatment and control in F1 generation 365 dpf.

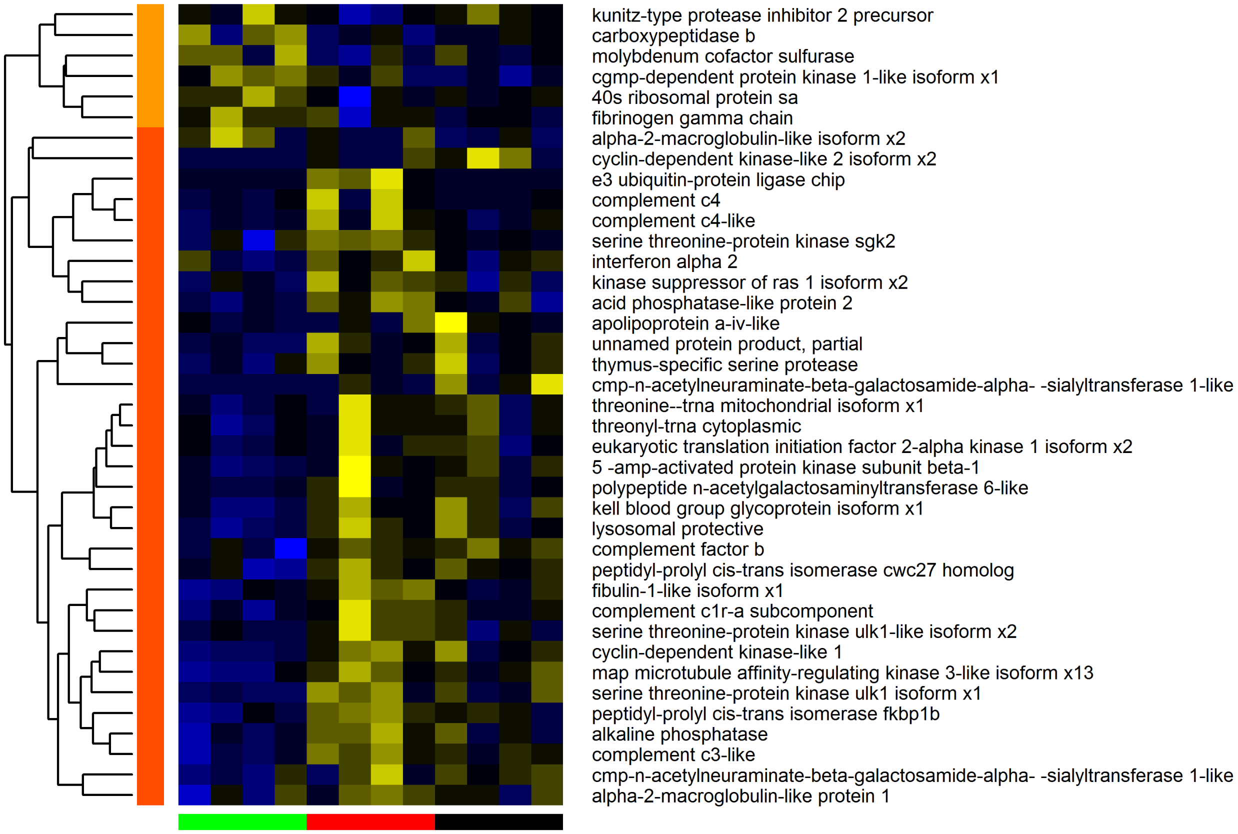

Supplementary Figure 2. Heatmap for genes related to protein metabolic process and differentially expressed between at least one BPA treatment and control in F2 generation 112 dpf.

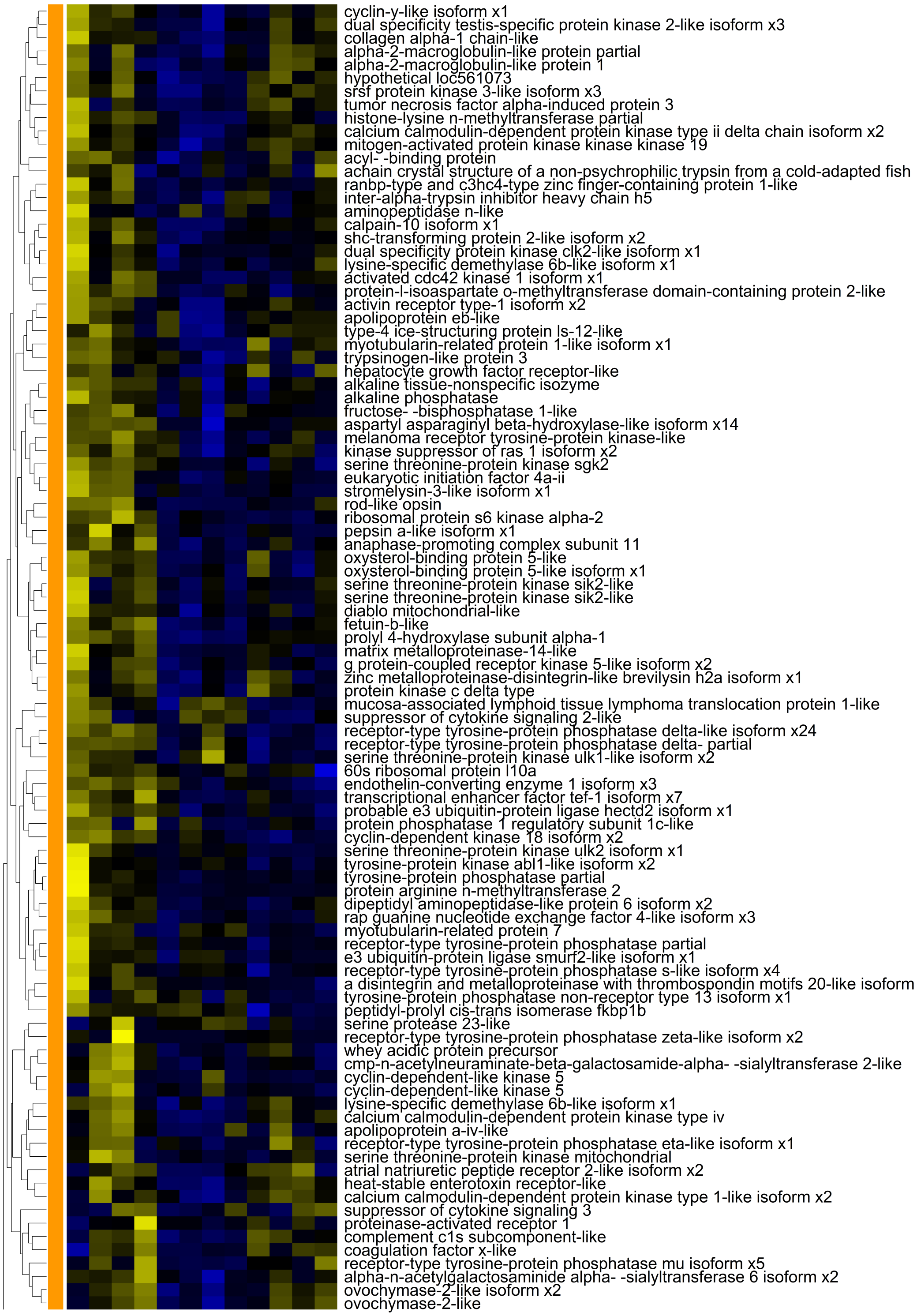
cyclin-y-like isoform x1
dual specificity testis-specific protein kinase 2-like isoform x3
collagen alpha-1 chain-like
alpha-2-macroglobulin-like protein partial
alpha-2-macroglobulin-like protein 1
hypothetical loc561073
srsf protein kinase 3-like isoform x3
tumor necrosis factor alpha-induced protein 3
histone-lysine n-methyltransferase partial
calcium calmodulin-dependent protein kinase type ii delta chain isoform x2
mitogen-activated protein kinase kinase kinase 19
acyl- -binding protein
achain crystal structure of a non-psychrophilic trypsin from a cold-adapted fish
ranbp-type and c3hc4-type zinc finger-containing protein 1-like
inter-alpha-trypsin inhibitor heavy chain h5
aminopeptidase n-like
calpain-10 isoform x1
shc-transforming protein 2-like isoform x2
dual specificity protein kinase clk2-like isoform x1
lysine-specific demethylase 6b-like isoform x1
activated cdc42 kinase 1 isoform x1
protein-l-isoaspartate o-methyltransferase domain-containing protein 2-like
activin receptor type-1 isoform x2
apolipoprotein eb-like
type-4 ice-structuring protein ls-12-like
myotubularin-related protein 1-like isoform x1
trypsinogen-like protein 3
hepatocyte growth factor receptor-like
alkaline tissue-nonspecific isozyme
alkaline phosphatase
fructose- -bisphosphatase 1-like
aspartyl asparaginyl beta-hydroxylase-like isoform x14
melanoma receptor tyrosine-protein kinase-like
kinase suppressor of ras 1 isoform x2
serine threonine-protein kinase sgk2
eukaryotic initiation factor 4a-ii
stromelysin-3-like isoform x1
rod-like opsin
ribosomal protein s6 kinase alpha-2
pepsin a-like isoform x1
anaphase-promoting complex subunit 11
oxysterol-binding protein 5-like
oxysterol-binding protein 5-like isoform x1
serine threonine-protein kinase sik2-like
serine threonine-protein kinase sik2-like
diablo mitochondrial-like
fetuin-b-like
prolyl 4-hydroxylase subunit alpha-1
matrix metalloproteinase-14-like
g protein-coupled receptor kinase 5-like isoform x2
zinc metalloproteinase-disintegrin-like brevilysin h2a isoform x1
protein kinase c delta type
mucosa-associated lymphoid tissue lymphoma translocation protein 1-like
suppressor of cytokine signaling 2-like
receptor-type tyrosine-protein phosphatase delta-like isoform x24
receptor-type tyrosine-protein phosphatase delta- partial
serine threonine-protein kinase ulk1-like isoform x2
60s ribosomal protein l10a
endothelin-converting enzyme 1 isoform x3
transcriptional enhancer factor tef-1 isoform x7
probable e3 ubiquitin-protein ligase hectd2 isoform x1
protein phosphatase 1 regulatory subunit 1c-like
cyclin-dependent kinase 18 isoform x2
serine threonine-protein kinase ulk2 isoform x1
tyrosine-protein kinase abl1-like isoform x2
tyrosine-protein phosphatase partial
protein arginine n-methyltransferase 2
dipeptidyl aminopeptidase-like protein 6 isoform x2
rap guanine nucleotide exchange factor 4-like isoform x3
myotubularin-related protein 7
receptor-type tyrosine-protein phosphatase partial
e3 ubiquitin-protein ligase smurf2-like isoform x1
receptor-type tyrosine-protein phosphatase s-like isoform x4
a disintegrin and metalloproteinase with thrombospondin motifs 20-like isoform
tyrosine-protein phosphatase non-receptor type 13 isoform x1
peptidyl-prolyl cis-trans isomerase fkbp1b
serine protease 23-like
receptor-type tyrosine-protein phosphatase zeta-like isoform x2
whey acidic protein precursor
cmp-n-acetylneuraminate-beta-galactosamide-alpha- -sialyltransferase 2-like
cyclin-dependent-like kinase 5
cyclin-dependent-like kinase 5
lysine-specific demethylase 6b-like isoform x1
calcium calmodulin-dependent protein kinase type iv
apolipoprotein a-iv-like
receptor-type tyrosine-protein phosphatase eta-like isoform x1
serine threonine-protein kinase mitochondrial
atrial natriuretic peptide receptor 2-like isoform x2
heat-stable enterotoxin receptor-like
calcium calmodulin-dependent protein kinase type 1-like isoform x2
suppressor of cytokine signaling 3
proteinase-activated receptor 1
complement c1s subcomponent-like
coagulation factor x-like
receptor-type tyrosine-protein phosphatase mu isoform x5
alpha-n-acetylgalactosaminide alpha- -sialyltransferase 6 isoform x2
ovochymase-2-like isoform x2
ovochymase-2-like

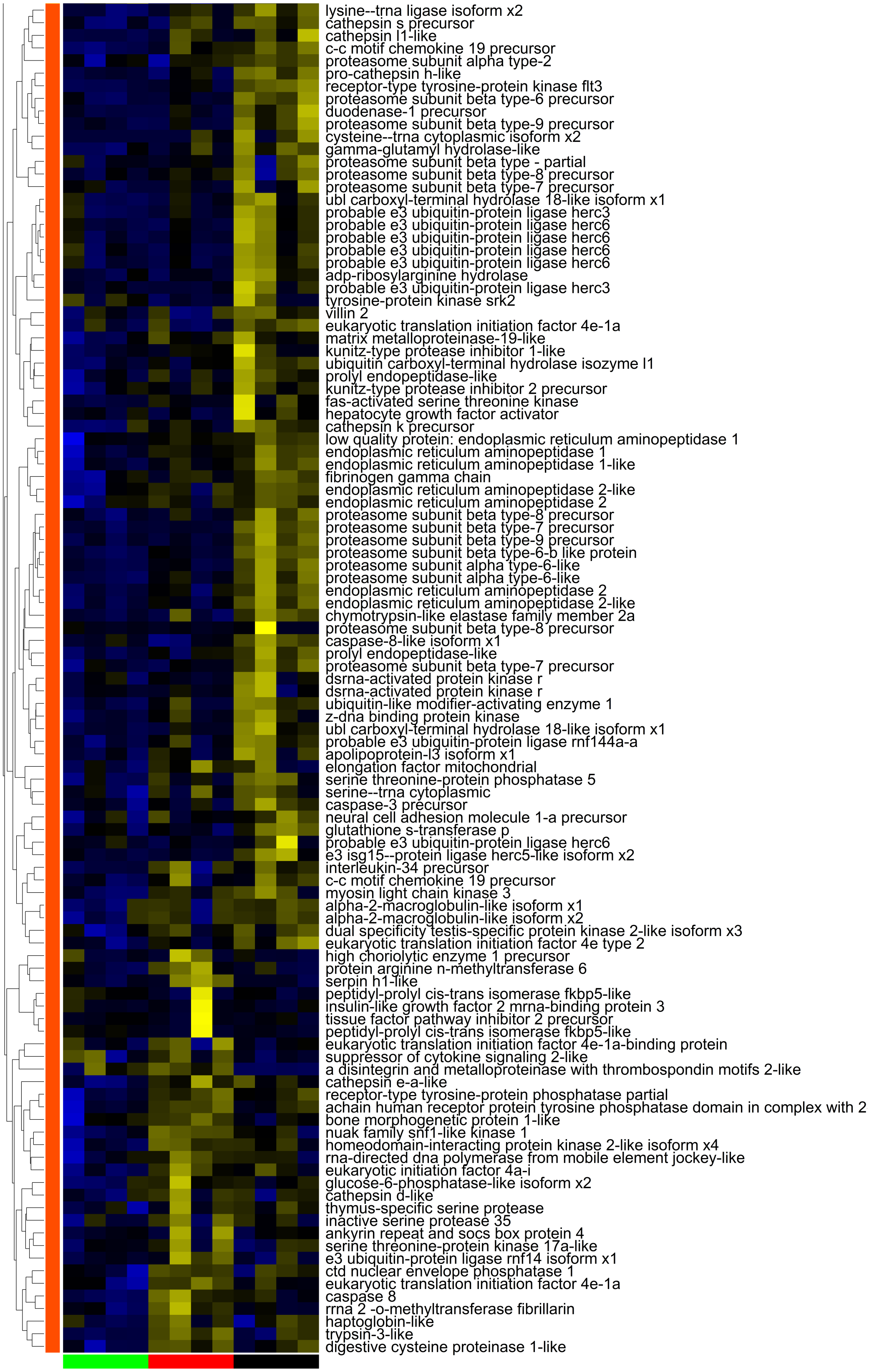

Supplementary Figure 3. Heatmap for genes related to protein metabolic process and differentially expressed between at least one BPA treatment and control in F2 generation 365 dpf.

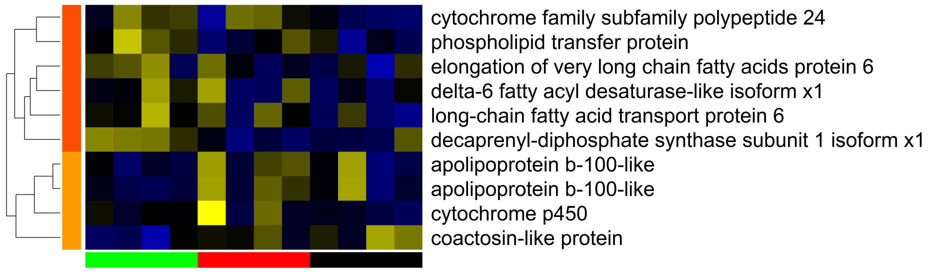

Supplementary Figure 4. Heatmap for genes related to lipid metabolic process and differentially expressed between at least one BPA treatment and control in F1 generation 140 dpf.

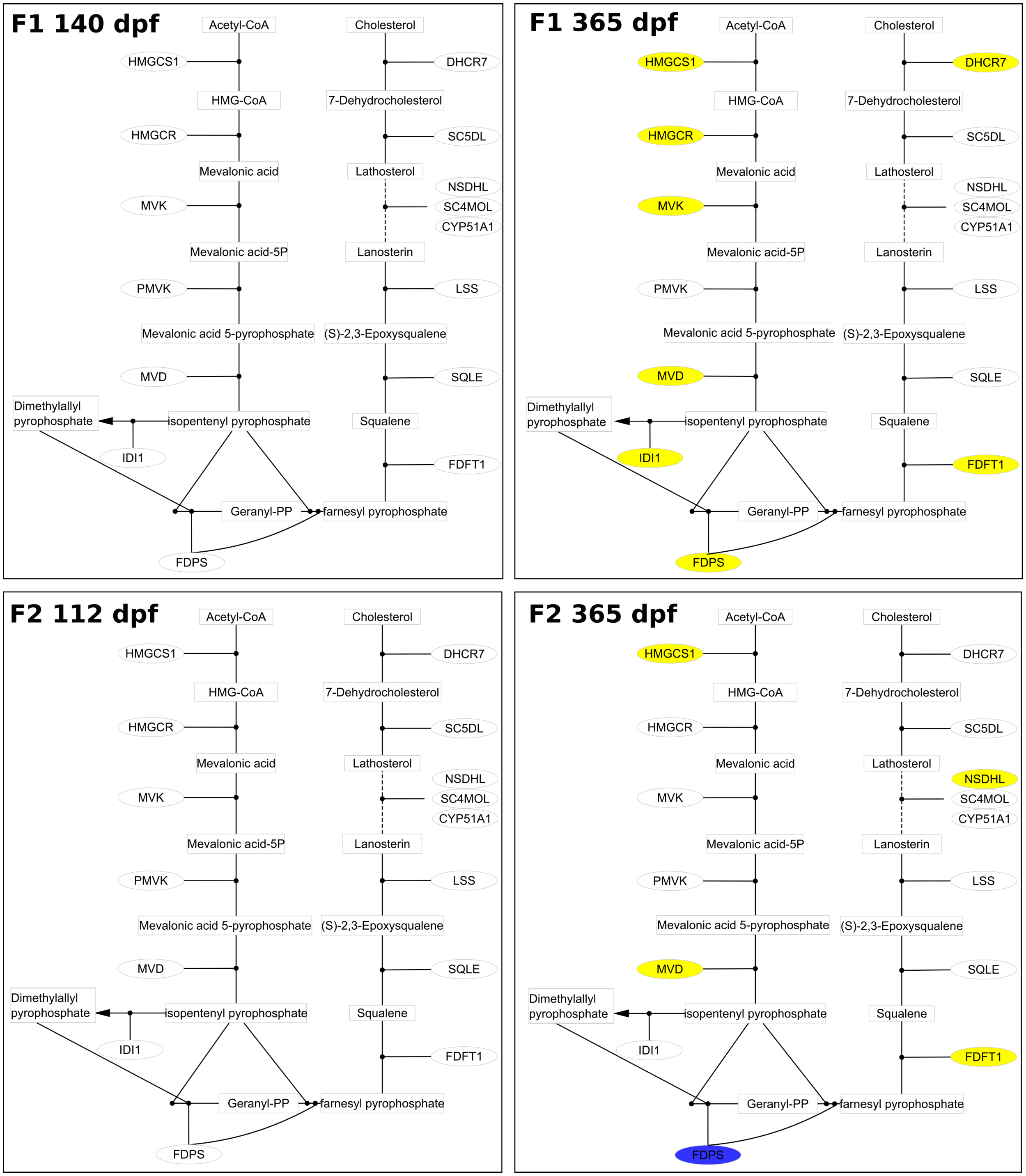

Supplementary Figure 5. BPA in rainbow trout eggs cause disruptions of the cholesterol biosynthesis pathway in juveniles (F1 365dpf and F2 365dpf), but not at early stages (F1 140dpf and F2 112dpf). Human's cholesterol biosynthesis pathway (WP197) was imported in Cytoscape. Genes upregulated and downregulated in fish raised from eggs containing 40 ng BPA are shaded yellow and blue, respectively. None of the genes from the cholesterol biosynthesis pathway was differentially expressed at early stages (F1 140 dpf and F2 112 dpf); CYP51A1 (cytochrome P450 family 51 subfamily A member 1), DHCR7 (7-dehydrocholesterol reductase), FDFT1 (farnesyl-diphosphate farnesyltransferase 1), FDPS (farnesyl

diphosphate synthase), HMGCR (3-hydroxy-3-methylglutaryl-CoA reductase), HMGCS1 (3-hydroxy-3-methylglutaryl-CoA synthase 1), IDI1 (isopentenyl-diphosphate delta isomerase 1), LSS (lanosterol synthase), MSMO1 (methylsterol monooxygenase 1), MVD (mevalonate diphosphate decarboxylase), MVK (mevalonate kinase), NSDHL (NAD(P) dependent steroid dehydrogenase-like), PMVK (phosphomevalonate kinase), SC5D (sterol-C5-desaturase), SQLE (squalene epoxidase)

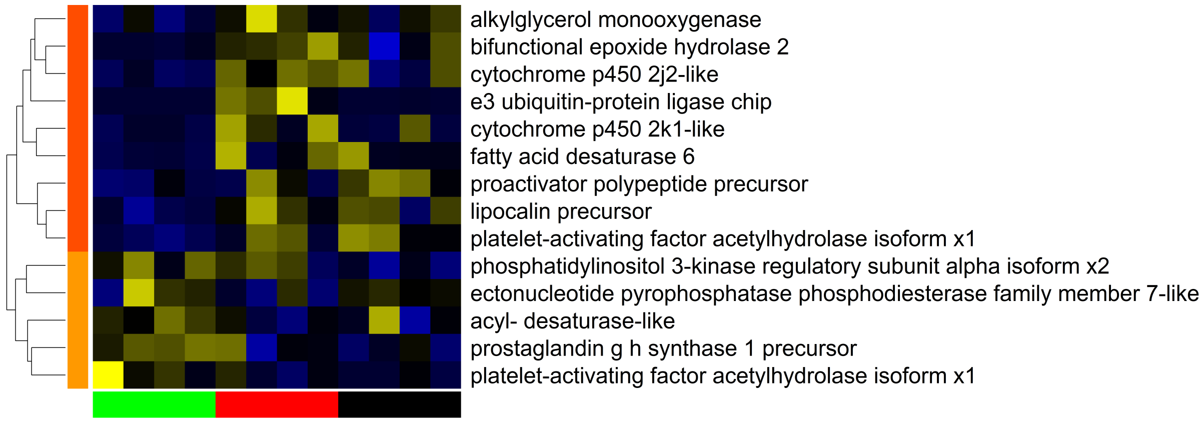

Supplementary Figure 6. Heatmap for genes related to lipid metabolic process and differentially expressed in at least one BPA treatment compared to control at 112 dpf in the F2 generation.

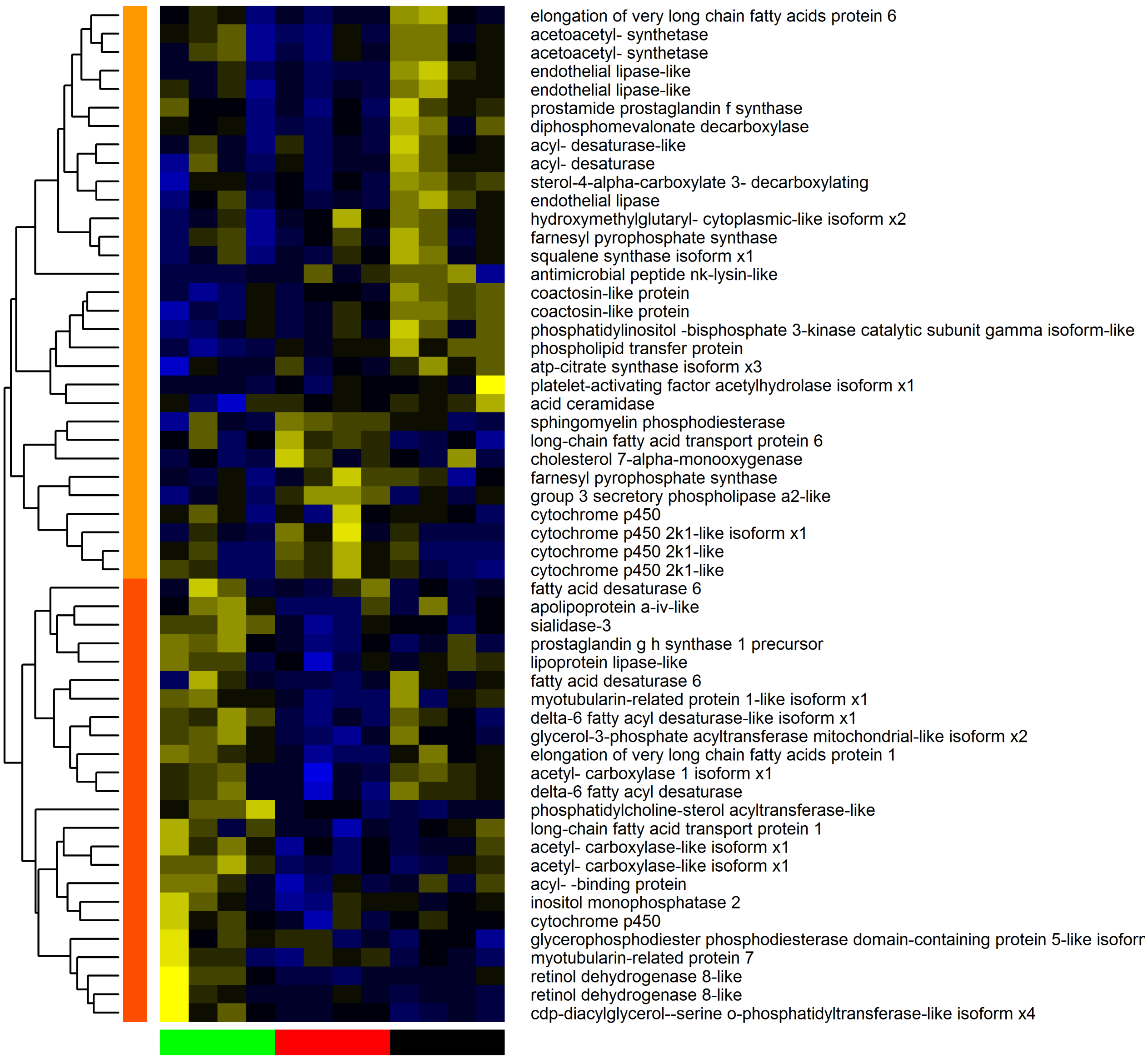

Supplementary Figure 7. Heatmap for genes related to lipid metabolic process and differentially expressed in at least one BPA treatment and control at 365 dpf in the F2 generation.
